# Sequenceserver: A Modern Graphical User Interface for Custom BLAST Databases

**DOI:** 10.1093/molbev/msz185

**Published:** 2019-08-14

**Authors:** Anurag Priyam, Ben J Woodcroft, Vivek Rai, Ismail Moghul, Alekhya Munagala, Filip Ter, Hiten Chowdhary, Iwo Pieniak, Lawrence J Maynard, Mark Anthony Gibbins, HongKee Moon, Austin Davis-Richardson, Mahmut Uludag, Nathan S Watson-Haigh, Richard Challis, Hiroyuki Nakamura, Emeline Favreau, Esteban A Gómez, Tomás Pluskal, Guy Leonard, Wolfgang Rumpf, Yannick Wurm

**Affiliations:** 1 School of Biological and Chemical Sciences, Queen Mary University of London, London, United Kingdom; 2 School of Chemistry and Molecular Biosciences, University of Queensland, Brisbane, Australia; 3 Department of Biotechnology, Indian Institute of Technology Kharagpur, Kharagpur, India; 4 Department of Mathematics, Indian Institute of Technology Kharagpur, Kharagpur, India; 5 Department of Computer Science, Royal Holloway University of London, Surrey, United Kingdom; 6 Scientific Computing Facility, Max Planck Institute of Molecular Cell Biology and Genetics, Dresden, Germany; 7 San Francisco, CA; 8 Computational Bioscience Research Center, King Abdullah University of Science and Technology, Thuwal, Kingdom of Saudi Arabia; 9 Bioinformatics Hub, School of Biological Sciences, University of Adelaide, Adelaide, Australia; 10 Institute of Evolutionary Biology, University of Edinburgh, Edinburgh, United Kingdom; 11 Spiber Inc, Kakuganji Tsuruoka, Yamagata, Japan; 12 Whitehead Institute for Biomedical Research, Cambridge, MA; 13 Living Systems Institute, University of Exeter, Exeter, United Kingdom; 14 The Institute for Genomic Medicine, The Abigail Wexner Research Institute at Nationwide Children’s Hospital, Columbus, OH; 15 5Bases Limited, London, United Kingdom; 16 Wellcome Sanger Institute, Wellcome Genome Campus, Cambridge, CB10 1SA

**Keywords:** visualization, BLAST, comparative genomics, sequence analysis

## Abstract

Comparing newly obtained and previously known nucleotide and amino-acid sequences underpins modern biological research. BLAST is a well-established tool for such comparisons but is challenging to use on new data sets. We combined a user-centric design philosophy with sustainable software development approaches to create Sequenceserver, a tool for running BLAST and visually inspecting BLAST results for biological interpretation. Sequenceserver uses simple algorithms to prevent potential analysis errors and provides flexible text-based and visual outputs to support researcher productivity. Our software can be rapidly installed for use by individuals or on shared servers.

## Introduction

The dramatic drop in sequencing costs has created many opportunities for individuals and groups of researchers to generate genomic or transcriptomic sequences from previously understudied organisms. Many research questions require small- or large-scale sequence comparisons, and BLAST (Basic Local Alignment Search Tool) is the most established tool for many such analyses (Altschul et al. 1990; Camacho et al. 2009). Unfortunately, BLAST analysis of new data can be challenging. There are delays before new data are submitted to and become publicly available on central BLAST repositories such as the NCBI (National Center for Biotechnology Information), and only small queries are feasible on such repositories. BLAST can be downloaded and installed locally, but its usage can be challenging for researchers without experience of command-line interfaces. Finally, commercial software to overcome such hurdles is too costly for many laboratories.

Here, we present Sequenceserver, a free graphical interface for BLAST designed to increase the productivity of biologist researchers performing and interpreting BLAST searches on custom data sets, and of bioinformaticians setting up shared laboratory or community databases. It has a user-centric focus (Garrett 2011) on accompanying researchers through their work process. Below, we provide an overview of Sequenceserver features that facilitate BLAST query submission and interpretation.

## Assisted Installation and BLAST Query Submission

**Fig. 1.**
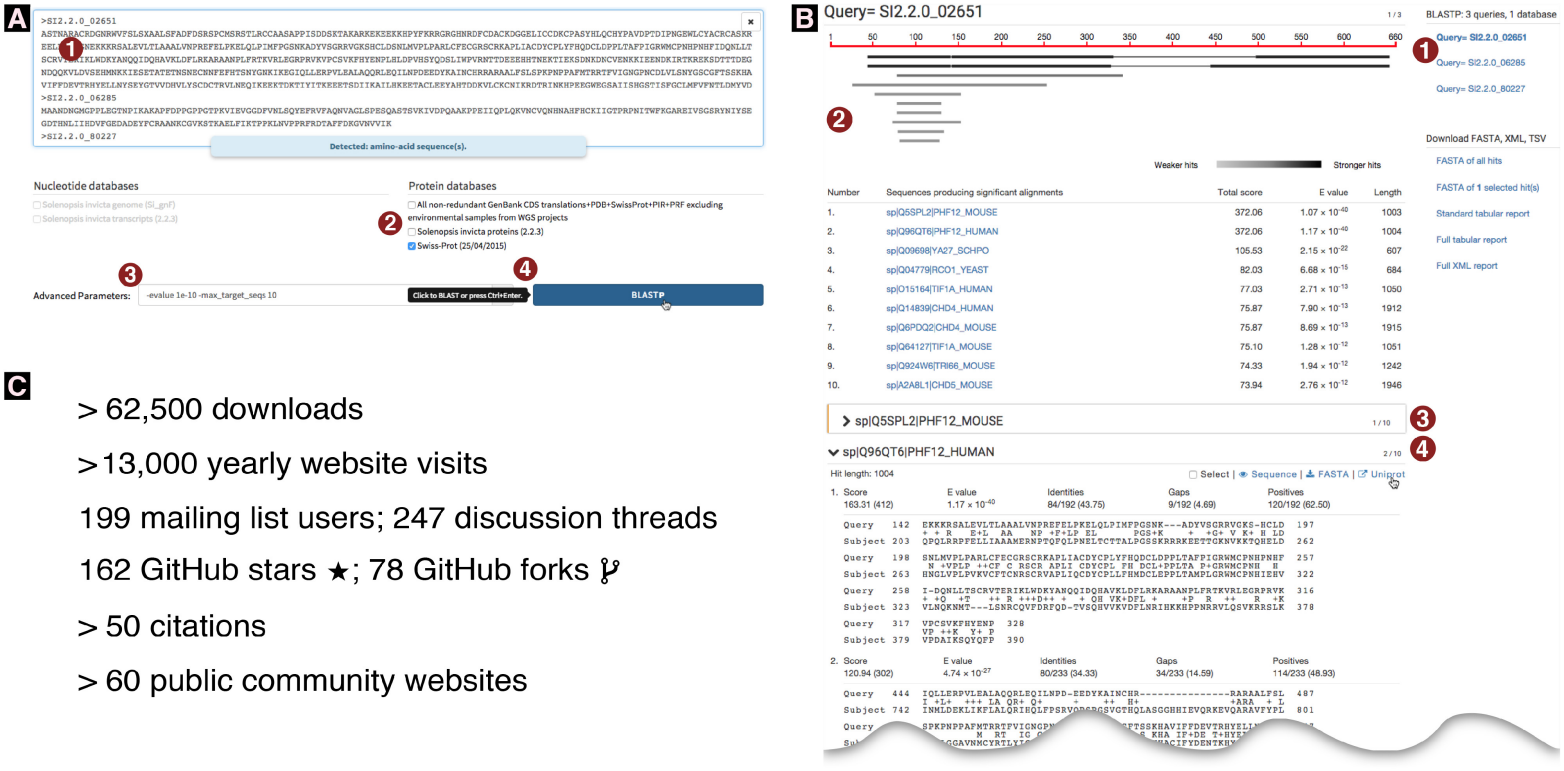
(*A*) Partial screenshot of the query interface. Numbers circled in red highlight the steps involved and some specific features. (1) Three or more sequences were pasted into the query field (typewriter font; only the identifier is visible for the third sequence); a message confirms to the user that these are amino acid sequences. (2) The Swiss-Prot protein database was the first database to be selected. As a result, additional database selections are limited to protein databases; nucleotide databases are disabled. (3) Optional advanced parameters were entered which constrain the results to the ten strongest hits with *E*-values stronger than 10^−10^. (4) The BLAST button is automatically activated and labeled “BlastP” as this is the only possible basic BLAST algorithm for the given query-database combination. As the user’s mouse pointer hovers over the BlastP button, a tooltip indicates that a keyboard shortcut exists for this button. (*B*) Partial screenshot of a Sequenceserver BLAST report. An interactive version of this figure is online at http://sequenceserver.com/paper/resultsinteractive (last accessed August 25, 2019). Three amino acid sequences were compared against the Swiss-Prot database using BlastP with an *E*-value cutoff of 10^−10^ and keeping only the ten strongest hits per query. This screenshot shows a portion of the results for the first query. Numbers circled in red highlight some specific features of this report. (1) An index overview summarizes the query and database information and provides clickable links to query-specific results. (2) Results for the first query are shown. These include a graphical overview indicating which parts of the query sequence align to each hit, a tabular summary of all hits, and alignment details for each hit. (3) The first hit is selected for download; its alignment details have been folded away. (4) The user is studying the second hit; the mouse pointer hovers over the link to the hit’s UniProt page. (*C*) Sequenceserver usage as of June 11, 2019. These include download statistics from https://rubygems.org/gems/sequenceserver, Google Analytics statistics for http://sequenceserver.com, and citation statistics from https://app.dimensions.ai/details/publication/pub.1085102830, and GitHub statistics from https://github.com/wurmlab/sequenceserver.

Installing Sequenceserver on computers running macOS or Linux is typically rapid, requiring only one or few commands (see online documentation). If necessary, Sequenceserver automates the download of BLAST (Camacho et al. 2009) binaries and can manage the conversion of FASTA files to BLAST databases. A user accesses Sequenceserver’s graphical interface in a web browser at http://localhost:4567 (fig. 1*A*). All detected BLAST databases are automatically listed here. The user types, pastes or drag-and-drops FASTA format query sequences into a text-field (fig. 1*A*). To prevent common errors, an alert message is shown and query submission is disabled if the query is invalid (e.g., combining nucleotide and protein sequences). The user then selects databases. The appropriate basic BLAST algorithm will automatically be used (supplementary fig. S1, Supplementary Material online). When multiple algorithms are appropriate, a pull-down in the BLAST submission button allows the user to toggle between them. An “advanced parameters” field provides access to all standard BLAST parameters.

## BLAST Result Visualization and Further Analysis

The Sequenceserver results page is designed to facilitate navigation, interpretation, and follow-up analysis (fig. 1*B* and http://sequenceserver.com/paper/resultsinteractive; last accessed August 25, 2018). Results are visually structured and will feel familiar to users of NCBI BLAST. If multiple query sequences were submitted, a clickable index of queries is shown. Queries, hits, and BLAST HSPs (high-scoring segment pairs) are numbered to facilitate navigation. For each query, identified hits are summarized in a table and an overview graphic. Each hit includes links for FASTA download, sequence visualization, and potentially to other resources. Such links can be automatically added based on regular expression analysis of identifiers (see online documentation). BLAST results can be downloaded in XML or tab-delimited table formats for further analysis. Similarly, a FASTA file containing all hit sequences, or a selection of hit sequences can be downloaded.

## Usage by Individual Researchers and as Part of Community Databases

Usage statistics including downloads, preprint citations, GitHub, and mailing list participation (fig. 1*C*) indicate that Sequenceserver is extensively used for molecular-genetic research on emerging model organisms (supplementary table S1, Supplementary Material online). For example, Sequenceserver installations on personal computers helped characterize the evolution of tunicate genomes (Blanchoud et al. 2018), fire ant olfactory genes (Pracana et al. 2017), and loci affecting Sorghum shoot architecture (McCormick et al. 2016). Sequenceserver has also been used to analyze human prostate cancer genomes (Seim et al. 2017) and to identify bacteria affecting shelf life of milk (Reichler et al. 2018).

Importantly, Sequenceserver also represents a main querying mechanism for more than 50 community genome databases (supplementary table S2, Supplementary Material online), including the PHI-base database of genes underpinning pathogen–host interactions (Winnenburg et al. 2006), an initiative to sequence 1,000 wild yeast genomes (Shen et al. 2016), and the http://reefgenomics.org coral genomics database; last accessed August 25, 2019 (Liew et al. 2016). Such community resources typically integrate Sequenceserver as part of larger web servers (e.g., Nginx [Reese 2008]) and customize it by adding links from BLAST hits to genome browsers or other gene-specific information. Additionally, many password-protected Sequenceserver instances exist for unpublished data.

## Outlook

In creating Sequenceserver, we aimed to respect user-centric design principles, open-source, and sustainable software engineering practices (Supplementary Material online). Our software is built using Ruby and Javascript frameworks commonly used for professional software development. The resulting robust architecture and flexibility facilitate customization and integration with other tools. This has led to contributions of improvements and bug-fixes by 21 bioinformaticians unrelated to the initial project; many are now coauthors. Our community is testing the ability to import preexisting BLAST or DIAMOND XML result files (Buchfink et al. 2015), and new manners of visualizing results (Wintersinger and Wasmuth 2015; Cui et al. 2016). Such efforts will continue to improve the ability of researchers to analyze and interpret genomic data.

## Data Availability

Source code is available under GNU Affero General Public License (AGPL) 3.0 at https://github.com/sequenceserver (last accessed August 25, 2019). Additional documentation is available online at http://sequenceserver.com (last accessed August 25, 2019).

## Supplementary Material

Supplementary materials are available at *Molecular Biology and Evolution* online.

## Supplementary Material

msz185_Supplementary_Data

## References

[msz185-B1] Altschul SF, Gish W, Miller W, Myers EW, Lipman DJ. 1990 Basic Local Alignment Search Tool. J Mol Biol. 215 3:403–410. 2231712 10.1016/S0022-2836(05)80360-2

[msz185-B2] Blanchoud S, Rutherford K, Zondag L, Gemmell NJ, Wilson MJ. 2018 *De novo* draft assembly of the *Botrylloides leachii* genome provides further insight into tunicate evolution. Sci Rep. 8 1:5518. 29615780 10.1038/s41598-018-23749-w PMC5882950

[msz185-B3] Buchfink B, Xie C, Huson DH. 2015 Fast and sensitive protein alignment using DIAMOND. Nat Methods. 12 1:59–60. 25402007 10.1038/nmeth.3176

[msz185-B4] Camacho C, Coulouris G, Avagyan V, Ma N, Papadopoulos J, Bealer K, Madden TL. 2009 BLAST+: architecture and applications. BMC Bioinformatics 10:421. 20003500 10.1186/1471-2105-10-421 PMC2803857

[msz185-B5] Cui Y, Chen X, Luo H, Fan Z, Luo J, He S, Yue H, Zhang P, Chen R. 2016 BioCircos.js: an interactive Circos JavaScript library for biological data visualization on web applications. Bioinformatics 32 11:1740–1742. 26819473 10.1093/bioinformatics/btw041

[msz185-B6] Garrett JJ. 2011 The elements of user experience: user-centered design for the Web and beyond. Berkeley (CA): New Riders.

[msz185-B7] Liew YJ, Aranda M, Voolstra CR. 2016 Reefgenomics.org—a repository for marine genomics data. Database 2016:baw152. 10.1093/database/baw152 PMC5199144 28025343

[msz185-B8] McCormick RF, Truong SK, Mullet JE. 2016 3D sorghum reconstructions from depth images identify QTL regulating shoot architecture. Plant Physiol. 172 2:823–834. 27528244 10.1104/pp.16.00948 PMC5047103

[msz185-B9] Pracana R, Levantis I, Martínez-Ruiz C, Stolle E, Priyam A, Wurm Y. 2017 Fire ant social chromosomes: differences in number, sequence and expression of odorant binding proteins. Evol Lett. 1 4:199–210. 30283649 10.1002/evl3.22 PMC6121795

[msz185-B10] Reese W. 2008 Nginx: the high-performance web server and reverse proxy. Linux J. 173:2.

[msz185-B11] Reichler S, Trmčić A, Martin N, Boor K, Wiedmann M. 2018 *Pseudomonas fluorescens* group bacterial strains are responsible for repeat and sporadic postpasteurization contamination and reduced fluid milk shelf life. J Dairy Sci. 101 9:7780. 29960782 10.3168/jds.2018-14438

[msz185-B12] Seim I, Jeffery PL, Thomas PB, Nelson CC, Chopin LK. 2017 Whole-genome sequence of the metastatic PC3 and LNCaP human prostate cancer cell lines. G3 (Bethesda) 7 6:1731–1741. 28413162 10.1534/g3.117.039909 PMC5473753

[msz185-B13] Shen XX, Zhou X, Kominek J, Kurtzman CP, Hittinger CT, Rokas A. 2016 Reconstructing the backbone of the *Saccharomycotina* yeast phylogeny using genome-scale data. G3 (Bethesda) 6 12:3927–3939. 27672114 10.1534/g3.116.034744 PMC5144963

[msz185-B14] Winnenburg R, Baldwin TK, Urban M, Rawlings C, Köhler J, Hammond-Kosack KE. 2006 PHI-base: a new database for pathogen host interactions. Nucleic Acids Res. 34(Database issue):D459–D464. 16381911 10.1093/nar/gkj047 PMC1347410

[msz185-B15] Wintersinger JA, Wasmuth JD. 2015 Kablammo: an interactive, web-based blast results visualizer. Bioinformatics 31 8:1305–1306. 25481007 10.1093/bioinformatics/btu808

