# Supplementary material for: Sequenceserver: A Modern Graphical User Interface for Custom BLAST Databases
Source: Mol Biol Evol. 2019 Aug 14;36(12):2922–4. doi: 10.1093/molbev/msz185 (PMC6878946; doi:10.1093/molbev/msz185)
Supplement: msz185_Supplementary_Data [file msz185_supplementary_data.pdf]

Supplementary Information. Sequenceserver: a modern graphical user interface for custom BLAST databases

Contents

Supplementary Text **1**
Technical implementation details . . . 1
Sustainable software development approach . . . 1
User centric design of graphical user interface . . . 2

Supplementary Figure and Tables **3**
Fig. S1: Automatic BLAST algorithm selection. . . . 3
Table S1: Research using Sequenceserver . . . 3
Table S2: Public community websites using Sequenceserver . . . 4

Supplementary Text

Technical implementation details

We developed Sequenceserver from scratch rather than basing our work on the NCBI's initial Perl/CGI wwwblast wrapper (Tao, 2006) to reduce technical debt (Lehman, 1980). The core of Sequenceserver is written in the Ruby language (Flanagan and Matsumoto, 2008) popular for creating websites (Ruby et al., 2013) and bioinformatics tools (Goto et al., 2010), while JavaScript and HTML/CSS are used for layout and interactions in the web browser. We use preexisting tools and libraries to facilitate development: The lightweight framework Sinatra (Harris and Haase, 2012) is used to create URL endpoints to load the search form and run BLAST searches from the browser. BLAST searches are delegated to the compiled command line version of BLAST (Camacho et al., 2009); we use Ox (https://github.com/ohler55/ox) to parse BLAST XML and create the HTML report. Underscore (http://underscorejs.org/), HTML5 Shiv (https://github.com/afarkas/html5shiv), jQuery (http://jquery.com), jQuery UI (http://jqueryui.com), Webshim (https://afarkas.github.io/webshim/demos), and Bootstrap (http://getbootstrap.com) libraries create a uniform scripting environment (for dynamic aspects of the user interface) and a consistent look-and-feel (for visual layout) across browsers. The d3 (http://d3js.org/) and BioJS (Gómez et al., 2013) libraries are used respectively for generating the graphical overview and the sequence viewing interface. Details regarding versions of the different software libraries are indicated in the source code repository at https://github.com/wurmlab/sequenceserver.

Sustainable software development approach

We followed six software engineering practices to facilitate and accelerate development while increasing robustness, improving the long-term sustainability of the software (Prlić and Procter, 2012; Wilson et al., 2014). First, we used an open source and agile development approach (Shore and Warden, 2007) involving frequent incremental improvements, peer review and frequent deployment on our servers and within the community. Second, we structured the software according to the object-oriented programming paradigm (Weisfeld, 2013) to cleanly separate different parts of code. Third, we followed two important software development principles: "don't repeat yourself" (DRY) leads to fewer lines of code and thus fewer bugs, and makes it easier to read and understand code than if similar commands are repeated in several places (Hunt and Thomas, 2000); "keep it simple, stupid" (KISS) reduces unnecessary complexity and thus lowers risks and leads to higher maintainability (Raymond, 2003). Fourth, we reuse widely established software packages and libraries (see above) to benefit from work done by others. This accelerates our work and reduces the amount of Sequenceserver-specific code, which in turn further reduces the likelihood of adding

bugs (Sametinger, 1997). Fifth, we implemented unit and integration tests (Ammann and Offutt, 2008) for many parts of Sequenceserver's code, and use continuous integration (https://travis-ci.org/) to ensure these tests are automatically run whenever a change is made to the code, thus increasing the likelihood and speed of detecting errors. Sixth, we use automatic code checkers including rubocop (https://github.com/bbatsov/rubocop) and w3 validator to ensure that our code respects relevant style guides and development principles. Such respect of style standards (*e.g.*, names of variables and methods, code structure and formatting) makes code more accessible to others than if we had chosen no or different conventions (Martin, 2008; Wurm, 2015). Finally, we use the Code Climate platform (http://codeclimate.com) for automated reviews of code quality.

User centric design of graphical user interface

To ensure a fluid user experience that increases researcher productivity, we designed Sequenceserver around eight modern user interface design principles. First, the interface contains only essential information to minimize distractions for the user. Second, the information is laid out in a clear and hierarchically structured manner. As part of this, we paid special attention to typography, using typefaces specifically designed for legibility and aesthetics on electronic devices (Roboto and Open Sans). Third, we used automation where possible to minimize the amount of decisions the user must make. For example, we limit the choices for algorithm selection based on query type and databases selection – this is because only a single basic BLAST algorithm is possible for all cases except for nucleotide-nucleotide search (Figure S1). Fourth, we use interactive visual feedback and cues for step-by-step discovery of the workflow. For example, the BLAST button remains disabled until the user has provided query sequence(s) and selected target databases. If the user tries to click the BLAST button while it is disabled, a tooltip indicates that a required input is missing. Similarly, the selection of protein databases is automatically disabled if the user has already selected a nucleotide database (and *vice versa*). Fifth, we remain consistent and contextual with regards to user interaction. For example, notification of detection of sequence type does not depend on how the query sequence was provided. This notification is shown below the query sequence input field – where the user is likely to look after query input – instead of using a global designated notification area or displaying pop-up windows that can be disruptive or are ignored. Similarly, a "clear query" button is shown only after the user has provided query sequence(s) and is positioned where a user is likely to look for it. Sixth, we try not to let the advantages of a graphical interface and efforts to create an easily accessible user experience limit the scope of what the user can do. For example, all possible advanced BLAST search options can be entered via a generic input field. Similarly, tooltips over report download links are only shown after the mouse pointer has hovered for at least 500ms. This delay means most users will not be bothered by tooltips after they have used the interface a few times. Seventh, we exploit intuitive human notions of colors. For example, if the user erroneously tries to combine nucleotide and amino acid sequences in the query, the query input-area is gently highlighted using a red border to indicate an error. At a different level, in the graphical overview shown for each query, the color of each hit indicates its strength, with stronger e-values being darker. Finally, the wording of error messages is similar to an informal human conversation to create empathy and familiarity, which may also clarify that Sequenceserver is built by a community of scientists.

Supplementary Figure and Tables

Figure S1. Automatic BLAST algorithm selection. BLAST includes five basic algorithms (right column). Arrows indicate how Sequenceserver automatically selects an appropriate BLAST algorithm based on the sequence types of the query (left column) and selected databases (middle column). For the first three combinations of query and database types, only one algorithm is possible. The circle indicates that for nucleotide query and nucleotide database, the user can choose between BLASTN and TBLASTX.

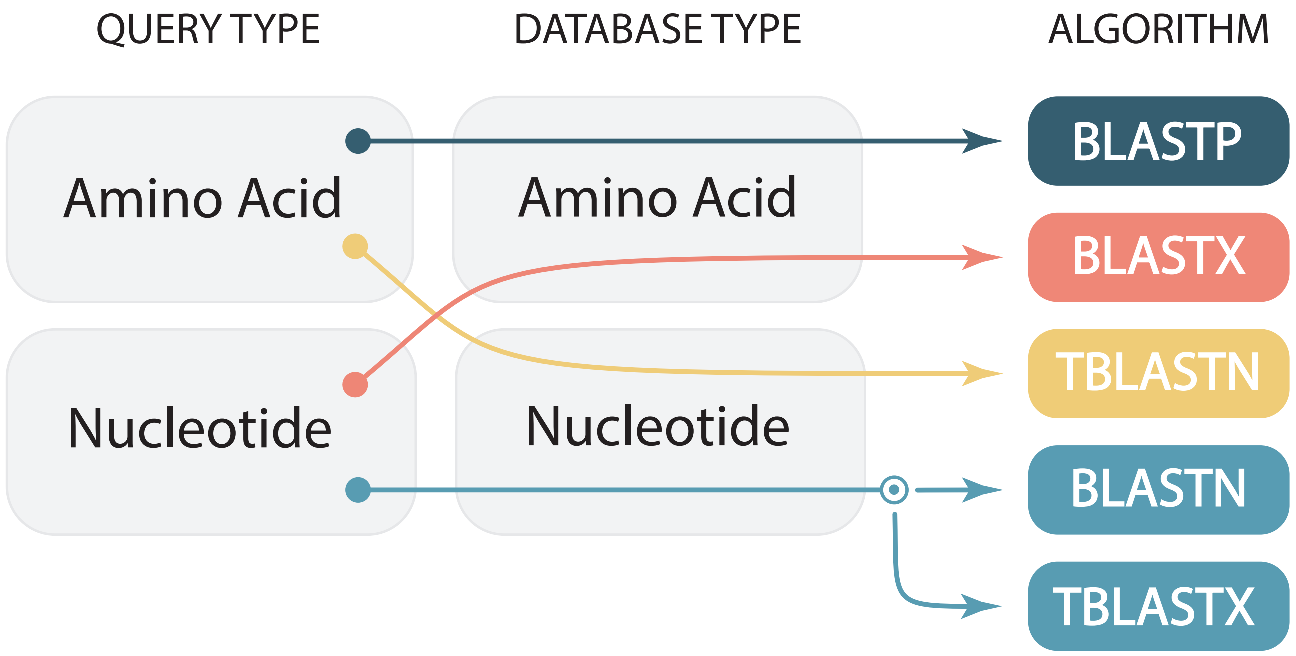

Table S1. Research using Sequenceserver

Interplay of chimeric mating-type loci impairs fertility rescue and accounts for intra-strain variability in *Zygosaccharomyces rouxii* interspecies hybrid ATCC42981	Bizzarri et al., 2019
A genome-wide association study of non-photochemical quenching in response to local seasonal climates in *Arabidopsis thaliana*	Rungrat et al., 2019
Taraxacum kok-saghyz (rubber dandelion) genomic microsatellite loci reveal modest genetic diversity and cross-amplify broadly to related species	Nowicki et al., 2019
Developmental expression and evolution of hexamerin and haemocyanin from *Folsomia candida* (Collembola)	Liang et al., 2019
Disentangling the mechanisms of mate choice in a captive koala population	Brandies et al., 2018
Evidence for sexual reproduction: Identification, frequency, and spatial distribution of *Venturia effusa* (pecan scab) mating type idiomorphs	Young et al., 2018
Pseudomonas fluorescens group bacterial strains are responsible for repeat and sporadic postpasteurization contamination and reduced fluid milk shelf life	Reichler et al., 2018
Complete pathway elucidation and heterologous reconstitution of *Rhodiola salidroside* biosynthesis	Torrens-Spence et al., 2018
Evolution of the shut-off steps of vertebrate phototransduction	Lamb et al., 2018

De novo draft assembly of the *Botrylloides leachii* genome provides further insight into tunicate evolution	Blanchoud et al., 2018
Whole-genome sequence of the metastatic PC3 and LNCaP human prostate cancer cell lines	Seim et al., 2017
Fire ant social chromosomes: Differences in number, sequence and expression of odorant binding proteins	Pracana et al., 2017
Ecological genomics for the conservation of dwarf birch.	Borrell, 2017
Transcriptomic discovery and comparative analysis of neuropeptide precursors in sea cucumbers (Holothuroidea)	Suwansa-ard et al., 2018
High-throughput genotyping analyses and image-based phenotyping in *Sorghum bicolor*	McCormick, 2017
Bacteriocins of non-aureus staphylococci isolated from bovine milk	Carson et al., 2017
Naturally occurring high oleic acid cottonseed oil: Identification and functional analysis of a mutant allele of *Gossypium barbadense* fatty acid desaturase-2	Shockey et al., 2016
3D sorghum reconstructions from depth images enable identification of quantitative trait loci regulating shoot architecture	McCormick et al., 2016
A workflow for studying specialized metabolism in nonmodel eukaryotic organisms	Torrens-Spence et al., 2016
Transcriptomic identification of starfish neuropeptide precursors yields new insights into neuropeptide evolution	Semmens et al., 2016
Multi-species sequence comparison reveals conservation of ghrelin gene-derived splice variants encoding a truncated ghrelin peptide	Seim et al., 2016
Characterization of a second secologanin synthase isoform producing both secologanin and secoxyloganin allows enhanced *de novo* assembly of a *Catharanthus roseus* transcriptome	Dugé de Bernonville et al., 2015
Identification and heterologous expression of the chaxamycin biosynthesis gene cluster from *Streptomyces leeuwenhoekii*	Castro et al., 2015
Discovery of sea urchin NGFFFamide receptor unites a bilaterian neuropeptide family	Semmens et al., 2015
Comparative analysis reveals loss of the appetite-regulating peptide hormone ghrelin in falcons	Seim et al., 2015
Reconstructing SALMFamide neuropeptide precursor evolution in the phylum Echinodermata: Ophiuroid and crinoid sequence data provide new insights	Elphick et al., 2015
Molecular biology approaches in bioadhesion research	Rodrigues et al., 2014
Discovery of a novel methanogen prevalent in thawing permafrost	Mondav et al., 2014
Neuropeptides and polypeptide hormones in echinoderms: New insights from analysis of the transcriptome of the sea cucumber *Apostichopus japonicus*	Rowe et al., 2014
Discovery of a novel neurophysin-associated neuropeptide that triggers cardiac stomach contraction and retraction in starfish	Semmens et al., 2013
The evolution and diversity of SALMFamide neuropeptides	Elphick et al., 2013
The protein precursors of peptides that affect the mechanics of connective tissue and/or muscle in the echinoderm *Apostichopus japonicus*	Elphick, 2012

Table S2. Public community websites using Sequenceserver

Reference / description	URL
Dieterich et al., 2007. Genomic resources for the nematode, Pristionchus pacificus	http://pristionchus.org

Amborella Genome Project, 2013. Amborella genome database	http://amborella.uga.edu
Chiu et al., 2013. Spotted wing fly-base	http://spottedwingflybase.org
Petrillo et al., 2015. JRC GMO-amplicons: Database of amplicon sequences related to genetically modified organisms	http://gmo-crl.jrc.ec.europa.eu/ jrcgmoamplicons/db_scans/blast
Kirmitzoglou and Promponas, 2015. LCR-eXXXplorer: Explore low complexity regions in protein sequences	http://repeat.biol.ucy.ac.cy/fgb2/ gbrowse/swissprot/
Brandl et al., 2016. Planmine: Data and tools to mine planarian biology	http://planmine.mpi-cbg.de
Mun et al., 2016. Lotus-base: Resources, tools, and datasets for the model legume Lotus japonicus	http://lotus.au.dk
Liew et al., 2016. ReefGenomics: Genomic and transcriptomic data for marine organisms	http://reefgenomics.org
Shen et al., 2016. Y1000+ project: Initiative to sequence 1000 wild yeasts	http://y1000plus.wei.wisc.edu
Nakagawa and Takahashi, 2016. gEVE: Database of genome-based endogenous viral elements	http://geve.med.u-tokai.ac.jp
Janies et al., 2016. EchinoDB: Database of orthologous transcripts from echinoderms	http://echinodb.uncc.edu
Louro et al., 2016. Assembled transcriptomes of sea bass and sea bream	http://sea.ccmar.ualg.pt:4567
Hane et al., 2016. Lupin genome portal: Genome assembly and annotations for the narrow-leafed lupin	http://lupinexpress.org
Challis et al., 2016. Lepbase: Lepidopteran genome database	http://lepbase.org
Zhu et al., 2017. CottonFGD: Cotton functional genomics database	http://cottonfgd.org
Hill et al., 2017. Hopbase: Database for genomics of Humulus lupulus (hop)	http://hopbase.org
Torres et al., 2017. LeishDB: Database for leishmania genomic information	http://leishdb.com
Naas et al., 2017. BLDB: Beta-lactamase database	http://bldb.eu:4567
Elsik et al., 2018. Hymenoptera genome database	http://hymenopteragenome.org
Hagen et al., 2018. Bovine genome database	http://bovinegenome.org
Meng et al., 2019. CircFunBase: A database for functional circular RNAs	http://bis.zju.edu.cn/CircFunBase/
Ravindran et al., 2018. Daphnia stressor database: Gene expression database for Daphnia	http://www.daphnia-stressordb. uni-hamburg.de/dsdbstart.php
Gene expression database for Alvinella pompejana, and Platynereis dumerilii	http://Jekely-lab.tuebingen.mpg.de
EFISH Genomics 2.0: web portal for electric fish genomic resources	http://efishgenomics. integrativebiology.msu.edu
NBIGV, Non-B cell derived immunoglobulin variable region database	http://nbigv.org
iBeetle-base: Database of Tribolium RNAi phenotypes	http://ibeetle-base.uni-goettingen.de
Cacao genome database	http://cacaogenomedb.org
Ant genomes, predicted transcripts and proteome	http://antgenomes.org
Aplysia transcriptome	http://aplysiagenetools.org:4567
Ash tree genome	http://ashgenome.org
Asparagus genome project	http://asparagus.uga.edu
Dwarf birch genome project	http://birchgenome.org
Fallon et al., 2018. Firefly genome database	http://blast.fireflybase.org
Genome, predicted transcripts and proteins of tardigrades	http://blast.tardigrades.org

Botulinum neurotoxin database	http://bontbase.org
eplant.org: Sequenced genomes of all plants to facilitate comparative genomic studies	http://eplant.org:4567
FusoPortal: A Fusobacterium genome and bioinformatic repository	http://fusoportal.org
NCHU fish genome database	http://lep-fish.nchu.edu.tw:4567
Fish genome database	http://brcwebportal.cos.ncsu.edu:4567
MarpolBase: Genome database for the common liverwort, Marchantia polymorpha	http://marchantia.info
MitoFun: A curated resource of complete fungal mitochondrial genomes	http://mitofun.biol.uoa.gr
Oat genome	http://oatgenomeproject.org
Spiny mouse transcriptome	http://spinymouse.erc.monash.edu
Measles, mumps, and rubella viruses database and analysis resource	http://mmrdb.org
Whole-genome sequence of the metastatic PC3 and LNCaP human prostrate cancer cell lines	http://ghrelinlab.org
10.1093/dnares/dsz003 Genome database for Iberian ribbed newt	http://inewt.nibb.ac.jp:8111
Crop genomics lab's BLAST server	http://plantgenomics.snu.ac.kr
Exome of Kronos durum wheat and Cadenza bread wheat mutants	http://wheat-tilling.com
Gene expression analysis and visualisation for wheat	http://wheat-expression.com
Fungal genomics	http://fungalgenomics.science.uu.nl
Stazione Zoologica Anton Dohrn	http://glossary-blast.bioinfo.szn.it
Georgia State University	http://db.cbn.gsu.edu:4568
Desplan Lab (*Drosophila* developmental biology)	http://desplan-lab.bio.nyu.edu
Commonwealth Scientific and Industrial Research Organisation	http://hieracium.csiro.au
Institute of Cytology and Genetics of Siberian Branch of the Russian Academy of Sciences	http://seqserver.sysbio.cytogen.ru
Taiwan Agricultural Genomics Resource Center	http://tagrc.org:4568, http://tagrc.org:4569

References

Amborella Genome Project (2013). The *Amborella* genome and the evolution of flowering plants. *Science* 342:1241089–1241089.

Ammann P and Offutt J (2008). *Introduction to software testing*. Cambridge University Press, New York.

Bizzarri M, Cassanelli S, Bartolini L, Pryszcz LP, Dušková M, Sychrová H, and Solieri L (2019). Interplay of chimeric mating-type loci impairs fertility rescue and accounts for intra-strain variability in *Zygosaccharomyces rouxii* interspecies hybrid ATCC42981. *Front Genet* 10.

Blanchoud S, Rutherford K, Zondag L, Gemmell NJ, and Wilson MJ (2018). *De novo* draft assembly of the *Botrylloides leachii* genome provides further insight into tunicate evolution. *Sci Rep* 8:5518.

Borrell S James (2017). Ecological genomics for the conservation of dwarf birch. *PhD Thesis* .

Brandies PA, Grueber CE, Ivy JA, Hogg CJ, and Belov K (2018). Disentangling the mechanisms of mate choice in a captive koala population. *PeerJ* 6:e5438.

Brandl H et al. (2016). PlanMine: A mineable resource of planarian biology and biodiversity. *Nucleic Acids Res* 44:D764–D773.

Camacho C et al. (2009). BLAST+: Architecture and applications. *BMC Bioinformatics* 10:421.

Carson DA, Barkema HW, Naushad S, and De Buck J (2017). Bacteriocins of non-aureus staphylococci isolated from bovine milk. *Appl Environ Microbiol* 83:e01015–17.

Castro JF et al. (2015). Identification and heterologous expression of the chaxamycin biosynthetic gene cluster from *Streptomyces leeuwenhoekii*. *Appl Environ Microbiol* 81:5820–5831.

Challis RJ, Kumar S, Dasmahapatra KKK, Jiggins CD, and Blaxter M (2016). Lepbase: The lepidopteran genome database .

Chiu JC et al. (2013). Genome of *Drosophila suzukii*, the spotted wing *Drosophila*. *G3* 3:2257–2271.

Dieterich C, Roeseler W, Sobetzko P, and Sommer RJ (2007). Pristionchus.org: A genome-centric database of the nematode satellite species pristionchus pacificus. *Nucleic Acids Research* 35:D498–D502.

Dugé de Bernonville T, Foureau E, Parage C, Lanoue A, Clastre M, Londono MA, Oudin A, Houillé B, Papon N, Besseau S, and et al (2015). Characterization of a second secologanin synthase isoform producing both secologanin and secoxyloganin allows enhanced *de novo* assembly of a catharanthus roseus transcriptome. *BMC Genomics* 16:619.

Elphick MR (2012). The protein precursors of peptides that affect the mechanics of connective tissue and/or muscle in the echinoderm apostichopus japonicus. *PLoS ONE* 7:e44492.

Elphick MR, Semmens DC, Blowes LM, Levine J, Lowe CJ, Arnone MI, and Clark MS (2015). Reconstructing SALMFamide neuropeptide precursor evolution in the phylum echinodermata: Ophiuroid and crinoid sequence data provide new insights. *Front Endocrinol* 6:2.

Elphick MR et al. (2013). The evolution and diversity of SALMFamide neuropeptides. *PLoS ONE* 8:e59076.

Elsik CG, Tayal A, Unni DR, Burns GW, and Hagen DE (2018). Hymenoptera genome database: Using hymenopteramine to enhance genomic studies of hymenopteran insects. *Eukaryotic Genomic Databases* 513–556.

Fallon TR, Lower SE, Chang CH, Bessho-Uehara M, Martin GJ, Bewick AJ, Behringer M, Debat HJ, Wong I, Day JC, and et al (2018). Firefly genomes illuminate parallel origins of bioluminescence in beetles. *eLife* 7.

Flanagan D and Matsumoto Y (2008). *The Ruby programming language*. O'Reilly Media, Sebastopol.

Gómez J et al. (2013). BioJS: An open source JavaScript framework for biological data visualization. *Bioinformatics* 29:1103–1104.

Goto N et al. (2010). BioRuby: Bioinformatics software for the Ruby programming language. *Bioinformatics* 26:2617–2619.

Hagen DE, Unni DR, Tayal A, Burns GW, and Elsik CG (2018). Bovine genome database: Tools for mining the *Bos taurus* genome. *Eukaryotic Genomic Databases* 211–249.

Hane JK, Ming Y, Kamphuis LG, Nelson MN, Garg G, Atkins CA, Bayer PE, Bravo A, Bringans S, Cannon S, and et al (2016). A comprehensive draft genome sequence for lupin (*Lupinus angustifolius*), an emerging health food: insights into plant-microbe interactions and legume evolution. *Plant Biotechnol J* 15:318–330.

Harris A and Haase K (2012). *Sinatra: Up and running*. O'Reilly Media, Sebastopol.

Hill ST, Sudarsanam R, Henning J, and Hendrix D (2017). Hopbase: A unified resource for *Humulus* genomics. *Database* 2017:bax009.

Hunt A and Thomas D (2000). *The pragmatic programmer: From journeyman to master*. Addison-Wesley, Boston.

Janies DA, Witter Z, Linchangco GV, Foltz DW, Miller AK, Kerr AM, Jay J, Reid RW, and Wray GA (2016). EchinoDB, an application for comparative transcriptomics of deeply-sampled clades of echinoderms. *BMC Bioinformatics* 17.

Kirmitzoglou I and Promponas VJ (2015). LCR-eXXXplorer: A web platform to search, visualize and share data for low complexity regions in protein sequences. *Bioinformatics* 31:2208–2210.

Lamb TD, Patel HR, Chuah A, and Hunt DM (2018). Evolution of the shut-off steps of vertebrate phototransduction. *Open Biol* 8:170232.

Lehman M (1980). Programs, life cycles, and laws of software evolution. *Proc IEEE* 68:1060–1076.

Liang Y, Xie W, and Luan Y (2019). Developmental expression and evolution of hexamerin and haemocyanin from *Folsomia candida* (Collembola). *Insect Mol Biol* 12585.

Liew YJ, Aranda M, and Voolstra CR (2016). Reefgenomics.org - a repository for marine genomics data. *Database* 2016:baw152.

Louro B, Marques JP, Power DM, and Canário AV (2016). Having a BLAST: Searchable transcriptome resources for the gilthead sea bream and the European sea bass. *Mar Genomics* 30:67–71.

Martin RC (2008). *Clean code: A handbook of agile software craftsmanship*. Prentice Hall, Upper Saddle River.

McCormick RF (2017). High-throughput genotyping analyses and image-based phenotyping in sorghum bicolor. *PhD Thesis* .

McCormick RF, Truong SK, and Mullet JE (2016). 3D sorghum reconstructions from depth images identify QTL regulating shoot architecture. *Plant Physiol* 172:823–834.

Meng X, Hu D, Zhang P, Chen Q, and Chen M (2019). Circfunbase: A database for functional circular rnas. *Database* 2019.

Mondav R et al. (2014). Discovery of a novel methanogen prevalent in thawing permafrost. *Nat Commun* 5:3212.

Mun T, Bachmann A, Gupta V, Stougaard J, and Andersen SU (2016). Lotus base: An integrated information portal for the model legume lotus japonicus. *Sci Rep* 6.

Naas T, Oueslati S, Bonnin RA, Dabos ML, Zavala A, Dortet L, Retailleau P, and Iorga BI (2017). Beta-lactamase database (bldb) – structure and function. *J Enzyme Inhib Med Chem* 32:917–919.

Nakagawa S and Takahashi MU (2016). gEVE: A genome-based endogenous viral element database provides comprehensive viral protein-coding sequences in mammalian genomes. *Database* 2016:baw087.

Nowicki M, Zhao Y, Boggess SL, Fluess H, Payá-Milans M, Staton ME, Houston LC, Hadziabdic D, and Trigiano RN (2019). *Taraxacum kok-saghyz* (rubber dandelion) genomic microsatellite loci reveal modest genetic diversity and cross-amplify broadly to related species. *Sci Rep* 9.

Petrillo M, Angers-Loustau A, Henriksson P, Bonfini L, Patak A, and Kreysa J (2015). JRC GMO-amplicons: A collection of nucleic acid sequences related to genetically modified organisms. *Database* 2015:bav101.

Pracana R, Levantis I, Martínez-Ruiz C, Stolle E, Priyam A, and Wurm Y (2017). Fire ant social chromosomes: Differences in number, sequence and expression of odorant binding proteins. *Evolution Letters* 1:199–210.

Prlić A and Procter JB (2012). Ten simple rules for the open development of scientific software. *PLoS Comput Biol* 8:e1002802.

Ravindran SP, Lueneburg J, Gottschlich L, Tams V, and Cordellier M (2018). *Daphnia* stressor database: Taking advantage of a decade of *Daphnia* "-omics" data for gene annotation .

Raymond ES (2003). *The art of Unix programming*. Addison-Wesley, Boston.

Reichler S, Trmčić A, Martin N, Boor K, and Wiedmann M (2018). *Pseudomonas fluorescens* group bacterial strains are responsible for repeat and sporadic postpasteurization contamination and reduced fluid milk shelf life. *J Dairy Sci* 101:7780.

Rodrigues M et al. (2014). Molecular biology approaches in bioadhesion research. *Beilstein J Nanotechnol* 5:983–993.

Rowe ML et al. (2014). Neuropeptides and polypeptide hormones in echinoderms: New insights from analysis of the transcriptome of the sea cucumber *Apostichopus japonicus*. *Gen Comp Endocrinol* 197:43–55.

Ruby S et al. (2013). *Agile web development with Rails 4*. Pragmatic Bookshelf, Raleigh, 4th edition.

Rungrat T, Almonte AA, Cheng R, Gollan PJ, Stuart T, Aro EM, Borevitz JO, Pogson B, and Wilson PB (2019). A genome-wide association study of non-photochemical quenching in response to local seasonal climates in *Arabidopsis thaliana*. *Plant Direct* 3:e00138.

Sametinger J (1997). *Software engineering with reusable components*. Springer, New York.

Seim I, Jeffery PL, Thomas PB, Nelson CC, and Chopin LK (2017). Whole-genome sequence of the metastatic pc3 and lncap human prostate cancer cell lines. *G3* 7:1731–1741.

Seim I, Jeffery PL, Thomas PB, Walpole CM, Maugham M, Fung JNT, Yap PY, O'Keeffe AJ, Lai J, Whiteside EJ, and et al (2016). Multi-species sequence comparison reveals conservation of ghrelin gene-derived splice variants encoding a truncated ghrelin peptide. *Endocrine* 52:609–617.

Seim I et al. (2015). Comparative analysis reveals loss of the appetite-regulating peptide hormone ghrelin in falcons. *Gen Comp Endocrinol* 216:98–102.

Semmens DC, Mirabeau O, Moghul I, Pancholi MR, Wurm Y, and Elphick MR (2016). Transcriptomic identification of starfish neuropeptide precursors yields new insights into neuropeptide evolution. *Open Biol* 6:150224.

Semmens DC et al. (2013). Discovery of a novel neurophysin-associated neuropeptide that triggers cardiac stomach contraction and retraction in starfish. *J Exp Biol* 216:4047–4053.

Semmens DC et al. (2015). Discovery of sea urchin ngfffamide receptor unites a bilaterian neuropeptide family. *Open Biol* 5:150030.

Shen XX, Zhou X, Kominek J, Kurtzman CP, Hittinger CT, and Rokas A (2016). Reconstructing the backbone of the *Saccharomycotina* yeast phylogeny using genome-scale data. *G3* 6:3927–3939.

Shockey J, Dowd M, Mack B, Gilbert M, Scheffler B, Ballard L, Frelichowski J, and Mason C (2016). Naturally occurring high oleic acid cottonseed oil: Identification and functional analysis of a mutant allele of gossypium barbadense fatty acid desaturase-2. *Planta* 245:611–622.

Shore J and Warden S (2007). *The art of agile development*. O'Reilly, Sebastopol.

Suwansa-ard S, Chaiyamoon A, Talarovicova A, Tinikul R, Tinikul Y, Poomtong T, Elphick MR, Cummins SF, and Sobhon P (2018). Transcriptomic discovery and comparative analysis of neuropeptide precursors in sea cucumbers (holothuroidea). *Peptides* 99:231–240.

Tao T (2006). wwwblast: Setup and usage. http://www.ncbi.nlm.nih.gov/staff/tao/URLAPI/wwwblast/. Accessed 20 October 2015.

Torrens-Spence M, Fallon T, and Weng J (2016). A workflow for studying specialized metabolism in nonmodel eukaryotic organisms. *Synthetic Biology and Metabolic Engineering in Plants and Microbes Part B: Metabolism in Plants* 69–97.

Torrens-Spence MP, Liu CT, Pluskal T, Chung YK, and Weng JK (2018). Monoamine biosynthesis via a noncanonical calcium-activatable aromatic amino acid decarboxylase in psilocybin mushroom. *ACS Chem Biol* 13:3343–3353.

Torres F, Arias-Carrasco R, Caris-Maldonado JC, Barral A, Maracaja-Coutinho V, and De Queiroz ATL (2017). LeishDB: A database of coding gene annotation and non-coding rnas in *Leishmania braziliensis*. *Database* 2017:bax047.

Weisfeld M (2013). *The object-oriented thought process*. Addison-Wesley, Boston, 4th edition.

Wilson G et al. (2014). Best practices for scientific computing. *PLoS Biol* 12:e1001745.

Wurm Y (2015). Avoid having to retract your genomics analysis. *The Winnower* 2:e143696.68941.

Young CA, Bock CH, Charlton ND, Mattupalli C, Krom N, Bowen JK, Templeton M, Plummer KM, and Wood BW (2018). Evidence for sexual reproduction: Identification, frequency, and spatial distribution of *Venturia effusa* (pecan scab) mating type idiomorphs. *Phytopathology* 108:837–846.

Zhu T, Liang C, Meng Z, Sun G, Meng Z, Guo S, and Zhang R (2017). CottonFGD: An integrated functional genomics database for cotton. *BMC Plant Biol* 17:101.
